# Differences in the Level of Physical Activity among Adolescents from Various European Countries

**Published:** 2018-04

**Authors:** Józef BERGIER, Pongrác ÁCS, Ferdinand SALONNA, Ján JUNGER

**Affiliations:** 1. Dept. of Tourism and Recreation, Pope John Paul II State School of Higher Education, Biała Podlaska, Poland; 2. Institute of Physiotherapy and Sport Sciences, University of Pécs, Pécs, Hungary; 3. Faculty of Physical Culture, Palacký University of Olomouc, Olomouc, Czech Republic; 4. Dept. of Physical Education, Pavol Jozef Šafárik University in Košice, Košice, Slovakia

## Dear Editor-in-Chief

Lifestyle exerts the greatest effect on human health, and one of the most important factors is a systematic physical activity. However, the present activity of school adolescents decreases with age, while an increase is observed in the sedentary life mode. Thus, there is a need for monitoring physical activity of school adolescents to able to determine the degree of the effectiveness of implementation of health education programmes in various countries.

While seeking the most objective methods for the assessment of physical activity, a group of researchers prepared International Physical Activity Questionnaire, in long and short versions, for the population aged 15–69 (1). The development of such an instrument enables comparison of the levels of activity of societies from various countries and continents and conducting studies over the years to determine the tendencies.

Studies among adolescents generally showed a poorly beneficial image of their physical activity, higher in boys than girls, with great discrepancies in the levels of this activity even within the same continent, as noted on an example of Europe.

In order to better recognize the physical activity of adolescents from the European continent, its assessment was performed using the long version of the IPAQ questionnaire among school adolescents aged 15–17 from four countries of the Visegrad Group (Czech Republic, Poland, Slovakia, Hungary). The study was conducted in 2015 and covered 2425 adolescents, including 1277 girls (52.7%) and 1148 boys (47.3%). After rejection of incorrectly completed questionnaires, the data concerning 1926 school adolescents; 1121 girls (58.2%) and 805 boys (41.8%) were used for analysis.

The adolescents and school headmasters have given written consent for participating in the research. The research was conducted under the project entitled “Physical and recreational activity and eating habits of the youth in the V4 countries” coordinated by Professor Józef Bergier. The project did not require the Ethics Committee consent.

### Level of physical activity and its domains

**Fig. 1:**
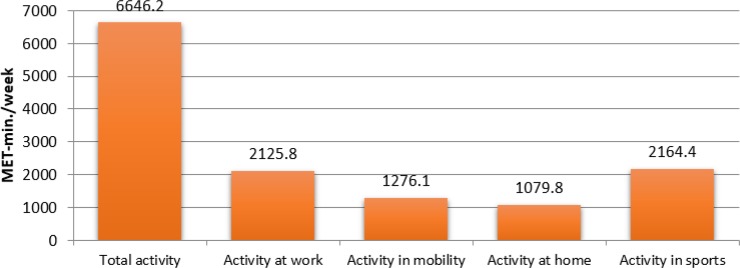
Levels and domains of physical activity of school adolescents in the countries of the Visegrad Group

**Fig. 2:**
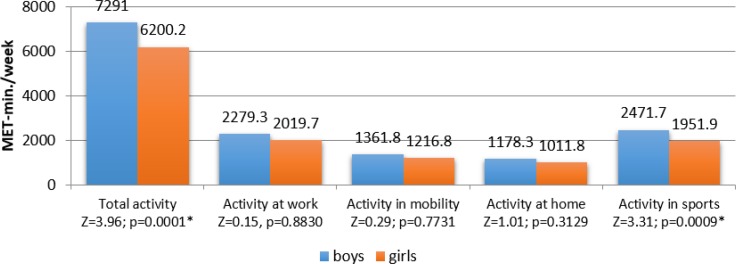
Level and domains of physical activity of school adolescents from the Visegrad Group countries, according to gender. // *Significant differences at *P*<0.05

The value of total physical activity expressed in the units’ MET-min/week among all adolescents in the study was 6646.2. The highest values were observed in the domain of sports activity (2164.4 MET) and at school (2125.8 MET), while the lowest values were noted for activity at home (1079.8 MET) and mobility–walking (1276.1 MET) (Fig. 1), and was significantly higher in boys −7291.0 MET-min/week, compared to girls −6200.2 MET-min/week; and in sports 1951.9 MET-min/week in girls and 2471.1 MET-min/week in boys (Fig. 2).

Significantly more beneficial result of physical activity among boys and girls was confirmed by compilations of the levels of this activity, where a high physical activity was characteristic of the majority of boys, rather than girls.

Studies of physical activity of adolescents from 4 European countries allow its positive assessment, i.e., higher than the studies conducted in other countries of Europe: Croatia (2), Lithuania (3) and Latvia (4). A similar relationship is also observed among students, to the benefit of males (5, 6).

Nevertheless, the subsequent studies, in this case of adolescents from the East European countries, indicate a considerably lower activity in girls than boys, confirmed by contemporary studies in various continents: America (7), Asia (8), 30 European countries, Israel, Canada, and the USA (9). Only explanation of the phenomenon by the ‘mobility laziness of girls’ is insufficient and requires the undertaking of new attractive programmes of physical activity for girls.
